# Supplementary material for: National trauma system establishment based on implementation of regional trauma centers improves outcomes of trauma care: A follow-up observational study in South Korea
Source: PLOS Glob Public Health. 2022 Jan 13;2(1):e0000162. doi: 10.1371/journal.pgph.0000162 (PMC10021375; doi:10.1371/journal.pgph.0000162)
Supplement: S2 Appendix — (PDF) [file pgph.0000162.s002.pdf]

S2 Appendix. Process of multi-panel review for preventable trauma death rate.

Process	Performers	Comparison of Number of Performers / Review Method	
		2015	2017
Pre-review	Trauma Coordinators	5	12
Moderate Pre-review Results & Assign Panels	Trauma Deaths Review Committee	5 Trauma Specialists (2 GS, 1 TS, 1 NS, and 1 EM)	
Individual Panel Review	Trauma Physicians	50	25
Team Panel Review	Multi-panel Teams	5 X 10 Teams*	5 X 5 Teams*
Review & Confirmation	Trauma Deaths Review Committee	5 Trauma Specialists (2 GS, 1 TS, 1 NS, and 1 EM)	
Reliability Test	Three Teams	3 Teams	3 Teams
Method to Review Medical Records	Pre-reviewers & Reviewers	• Reviewers had to visit the emergency medical institutions	• All data from the emergency medical institutions were reviewed in one place.

*One team comprised two general surgeons, one thoracovascular surgeon, one neurosurgeon, and one mergency physician.
GS, general surgeon; TS, thoracovascular surgeon; NS, neurosurgeon; EM, emergency physician.
