# Supplementary material for: National trauma system establishment based on implementation of regional trauma centers improves outcomes of trauma care: A follow-up observational study in South Korea
Source: PLOS Glob Public Health. 2022 Jan 13;2(1):e0000162. doi: 10.1371/journal.pgph.0000162 (PMC10021375; doi:10.1371/journal.pgph.0000162)
Supplement: S3 Appendix — (PDF) [file pgph.0000162.s003.pdf]

S3 Appendix. Summary of sampling method and estimation of traumatic deaths.

Item	Detailed Method	
**Population**	**Target Population**	▪ Trauma deaths who were transferred to the emergency medical institutions in South Korea
	**Investigation Population**	▪ Trauma deaths in emergency medical institutions which have more than one trauma death per year
**Sampling Unit**	**Primary Sampling Unit**	▪ Emergency medical institutions
	**Secondary Sampling Unit**	▪ Trauma deaths in emergency medical institutions
**Stratification**	**First Stratification Variable (Stratification of** Emergency **Medical Institutions)**	▪ Region (5): Seoul, Incheon/Gyeonggi, Daejeon/Chungcheong/Gangwon, Gwangju/Jeolla/Jeju, Busan/Daegu/Ulsan/Gyeongsang<br>▪ Level (type) of emergency medical institutions (3): REMC/RTC, LEMC, LEMI<br>▪ Number of trauma deaths (3): ≤9, 10–29, ≥30
	**Second Stratification Variable (Stratification of Death)**	▪ Place (timing) of trauma deaths (3): DOA, At emergency department, After hospitalization<br>▪ Patients' age (3): ≤14, 15–54, ≥55
**Sample Size**	▪ We initially calculated a total sample size of 1,000 for 2015 and 1,300 for 2017 after reviewing the sample size according to the level of target error. However, when the cases that were excluded from the panel review were included, the survey sample size was determined to be 1,131 and 1,862 for 2015 and 2017, respectively.<br>▪ This sample size was expected to meet the limit of error of approximately ±4.5% in 2015 and ±3.8% in 2017 at 95% confidence levels.	
**Sampling**	▪ The sample emergency medical institutions were extracted by stratified two-stage cluster random sampling.<br>▪ The sample trauma deaths were extracted by stratified random sampling, totaling 1,131 in 2015 and 1,862 in 2017.	
**Calculation of Sample Weight**	▪ To estimate the PTDR of the population, the sample weight of each hospital level and death was calculated according to the sampling method and applied to analyze the sample-designed survey data.	
**Estimation**	▪ Calculation of the parameter of interest (preventable trauma death rate) by the weighted estimates using design weights.<br>▪ Calculation of the standard error and the margin of error for the estimate of the parameter of interest (preventable trauma death rate).	

REMC, Regional Emergency Medical Center; RTC, Regional Trauma Center; LEMC, Local Emergency Medical Center; LEMI, Local Emergency Medical Institution; DOA, dead on arrival.
