# Supplementary material for: National trauma system establishment based on implementation of regional trauma centers improves outcomes of trauma care: A follow-up observational study in South Korea
Source: PLOS Glob Public Health. 2022 Jan 13;2(1):e0000162. doi: 10.1371/journal.pgph.0000162 (PMC10021375; doi:10.1371/journal.pgph.0000162)
Supplement: Attachment — Submitted filename: Response to Reviewers.docx [file pgph.0000162.s005.docx]

Response to Reviewers

(PGPH-D-21-00240) National trauma system establishment based on implementation of regional trauma centers improves outcomes of trauma care: A follow-up observational study in South Korea

- We sincerely appreciate the careful review of our manuscript and the helpful suggestions provided, which have contributed considerably to its improvement. The manuscript has been revised as suggested and detailed responses to the individual comments have been enclosed (The responses and the revised part of the manuscript was highlighted in yellow).

Additional Editor Comments (if provided):

A few minor concerns are as follows-

1. The data presented is from 2015 to 2017, but the system was established in 2012. Why not present the data from 2012 to 2020?

- We apologize for the confusion. A master plan with the aim of establishing 17 regional trauma centers nationwide in South Korea was developed in 2012, and 2 to 4 trauma centers have been opened every year since 2013. By 2017, although 16 centers had been designated, only 9 were officially operational. We also expected that it would take 2 years for the EMS and EMIs to adjust to the new trauma center policies and implement the new system. Accordingly, since 2015, we have planned a national follow-up survey on PTDR every 2 years following the implementation of the NTS to confirm its effectiveness. This manuscript reports on the results of the first survey conducted in 2015 and the second survey conducted in 2017.
- In addition, data for trauma patients, including trauma deaths, are only available after at least one year. It also takes nearly an additional year from sampling to analysis of the panel review results. We also wish to highlight that the third investigation for the 2019 trauma deaths is currently underway. At the time this study was conducted, we could extract data on trauma patients from 2013 to 2019; however, we preferred to focus on the 2015 and 2017 data only because we aimed to examine the changes in trauma performance and outcomes according to the change in preventable trauma death rates.

2. Were any weighted analyses conducted? Why not? How was power analyses conducted for sample size? based on what effects?

- Thank you for your questions. In this study, we initially calculated a total sample size of 1,000 for 2015 and 1,300 for 2017 after reviewing the sample size according to the level of target error. However, when the cases that were excluded from the panel review were included, the survey sample size was determined to be 1,131 and 1,862 for 2015 and 2017, respectively. This sample size was expected to meet the limit of error of approximately ±4.5% in 2015 and ±3.8% in 2017 at 95% confidence levels. To estimate the PTDR of the population, the sample weight of each hospital level and cause of death was calculated according to the sampling method and applied to analyze the sample-designed survey data.
- To improve our readers’ understanding of these calculations, the relevant details were added as a table in the Supporting Information (S3 Appendix. Summary of sampling method and estimation of traumatic deaths).

**S3 Appendix. Summary of sampling method and estimation of traumatic deaths.**

| **Item** | **Detailed Method** | |
| --- | --- | --- |
| **Population** | **Target Population** | ▪ Trauma deaths who were transferred to the emergency medical institutions in South Korea |
|  | **Investigation Population** | ▪ Trauma deaths in emergency medical institutions which have more than one trauma death per year |
| **Sampling Unit** | **Primary Sampling Unit** | ▪ Emergency medical institutions |
|  | **Secondary Sampling Unit** | ▪ Trauma deaths in emergency medical institutions |
| **Stratification** | **First Stratification Variable**  **(Stratification of** Emergency **Medical Institutions)** | ▪ Region (5): Seoul, Incheon/Gyeonggi, Daejeon/Chungcheong/Gangwon, Gwangju/Jeolla/Jeju, Busan/Daegu/Ulsan/Gyeongsang  ▪ Level (type) of emergency medical institutions (3): REMC/RTC, LEMC, LEMI  ▪ Number of trauma deaths (3): ≤9, 10–29, ≥30 |
|  | **Second Stratification Variable**  **(Stratification of Death)** | ▪ Place (timing) of trauma deaths (3): DOA, At emergency department, After hospitalization  ▪ Patients’ age (3): ≤14, 15–54, ≥55 |
| **Sample Size** | ▪ We initially calculated a total sample size of 1,000 for 2015 and 1,300 for 2017 after reviewing the sample size according to the level of target error. However, when the cases that were excluded from the panel review were included, the survey sample size was determined to be 1,131 and 1,862 for 2015 and 2017, respectively.  ▪ This sample size was expected to meet the limit of error of approximately ±4.5% in 2015 and ±3.8% in 2017 at 95% confidence levels. | |
| **Sampling** | ▪ The sample emergency medical institutions were extracted by stratified two-stage cluster random sampling.  ▪ The sample trauma deaths were extracted by stratified random sampling, totaling 1,131 in 2015 and 1,862 in 2017. | |
| **Calculation of**  **Sample Weight** | ▪ To estimate the PTDR of the population, the sample weight of each hospital level and death was calculated according to the sampling method and applied to analyze the sample-designed survey data. | |
| **Estimation** | ▪ Calculation of the parameter of interest (preventable trauma death rate) by the weighted estimates using design weights.  ▪ Calculation of the standard error and the margin of error for the estimate of the parameter of interest (preventable trauma death rate). | |

REMC, Regional Emergency Medical Center; RTC, Regional Trauma Center; LEMC, Local Emergency Medical Center; LEMI, Local Emergency Medical Institution; DOA, dead on arrival.

3. The manuscript at times runs like a long thesis and such redundancy should be cut down.

- We apologize for this. We have shortened our article accordingly by revising the Introduction and Materials and Methods.

4. When you mention Korea- replace it with South Korea throughout the paper.

- Thank you for this pertinent comment. We have changed all instances of Korea to South Korea in the revised manuscript.

5. What are the implications for practice? and future research?

- The purpose of this study was to evaluate the effects of the attempts to implement and establish a national trauma system (NTS) in South Korea, which has a high rate of preventable trauma mortality (>30%), comparable to that of low- and middle-income countries (LMICs). The South Korean government has continuously invested resources, including labor costs, to establish an NTS in cooperation with the health sector, which is a novel project that has not been conducted elsewhere. We aimed to conduct national surveys every two years to assess any reduction in the preventable trauma death rate, which was the original goal when the NTS was first developed at the end of 2012. In addition, the transfer status and outcome changes of trauma patients were investigated using the National Emergency Department Information System (NEDIS), which is utilized by over 400 emergency medical institutions nationwide. We observed that after the implementation of the NTS, severe trauma patients were more concentrated in the regional trauma centers; this subsequently led to a reduction in preventable trauma death rate and mortality of severe trauma patients.
- This study is meaningful in that it was conducted serially for all emergency medical institutions in South Korea. As this study was conducted in the field of traumatology, a completely prospective randomized controlled trial would not be possible; nevertheless, this study is a prospective observational study conducted serially from the time of the trauma system initiation. In addition, the results were derived by simultaneously conducting a preventable trauma death review (qualitative analysis) and a large dataset analysis (quantitative analysis).
- Evidence from this study and previous research indicates that trauma outcomes can improve through the establishment of trauma centers. In this study, severe trauma patients were more concentrated in the regional trauma centers and the preventable trauma death rate decreased by >10% in only 2 years after the establishment of the government-led trauma system, indicating that through national efforts trauma performance and outcomes can improve within a short timeframe after the trauma system is implemented.
- Furthermore, the overall performance and outcomes of trauma care improved in all emergency medical institutions nationwide. These findings provide evidence of the effectiveness of establishing a national trauma system and a protocol for assessing this effectiveness, especially in LMICs with a large burden of injury that do not hitherto have an effective trauma system.

6, There are many other limitations concerning reliability and validity – explain these and how would the limitations affect the results? What else could be done to improve the rigor of the study?

- Thank you for highlighting this important point. As stated in the WHO “Guidelines for trauma quality improvement programmes,” preventability judgements may be viewed as somewhat subjective, and there may be variation in reliability between different panels’ assessments in survey methodology, which is based on the death panel review. As a result, many limitations regarding reliability and validity are unavoidable but it is difficult to judge their impact. Nevertheless, we used this methodology because it remains a straightforward method of accomplishing the goal of assessing and improving the quality of care, as recommended by the WHO TQIP guidelines. In addition, since the goal of the NTS in South Korea was to reduce the preventable trauma death rate to less than 20%, this survey method was used as a basic framework.
- However, we following countermeasures were implemented to compensate for these limitations, and as a result, reliability for the panel review improved in 2017 compared to the first survey in 2015. First, we developed a review form following a checklist format and trained pre-reviewers and panelists before the review. Second, a review committee (super-panel) reviewed it once more to reduce the subjectivity of death panel reviews. Third, the 2015 and 2017 surveys were conducted in the same way and the majority of reviewers used were the same. Additionally, probability of survival models through the severity adjustment using injury severity scoring system from the National Emergency Department Information System (NEDIS) data was created to supplement the results of preventable death rate by panel reviews. The NEDIS is currently registered by over 400 emergency medical institutions (413 of 416 EMIs in 2017) nationwide in South Korea.
- These details have now been included in the Supporting Information (S1 Appendix. The structured review form including audit filters for the multi-panel review, S2 Appendix. Process of multi-panel review for preventable trauma death rate) of the revised manuscript.
