# Supplementary material for: National trauma system establishment based on implementation of regional trauma centers improves outcomes of trauma care: A follow-up observational study in South Korea
Source: PLOS Glob Public Health. 2022 Jan 13;2(1):e0000162. doi: 10.1371/journal.pgph.0000162 (PMC10021375; doi:10.1371/journal.pgph.0000162)
Supplement: Attachment — Submitted filename: ResponseToReviewers_211213.docx [file pgph.0000162.s006.docx]

**Response to Reviewers**

The manuscript has been carefully revised, and appropriate changes have been made based on the reviewers’ suggestions. We have also provided point-by-point responses to the reviewers’ comments. We thank you and the reviewers for your thoughtful suggestions and insights, which have enriched the manuscript and produced a more balanced account of our research.

**Reviewer #1:**

Major comments

1. This study actually compared the rates of two time points without using proper controls and this might be the major limitation of this study. If authors can show the results using negative controls to back up the causal interpretation of the study findings, it may increase the overall internal validity of the study.

**Response**:

Like the reviewer's opinion, we have attempted to conduct an appropriate comparative analysis, including setting appropriate negative controls. However, it was impossible to use negative controls to back up the causal interpretation of the study findings because we could not adjust injury severity appropriately due to the limited quality of information and data collected retrospectively for trauma death cases. Considering these points, we chose a method of judging directly through panel reviews, and quantitative analysis using big data analysis was added to compensate for the limitations of subjectivity resulting from this. Please understand these limitations. We apologize for not being able to fully reflect the reviewer's comment. We have summarized and added some of these in the limitation paragraph of the Discussion section.

2. Is there any other possible regulations, governmental policies, and societal changes which might affect the study findings other than implementation of regional trauma system? These contents should be reviewed and discussed in the manuscript.

**Response**:

We do not believe that any other possible regulations, governmental policies, and societal changes might affect the study findings other than the implementation of the regional trauma system. As mentioned in the main text, at the end of 2012, a master plan was made for the establishment of a national trauma system based on the implementation of 17 regional trauma centers in Korea. The development of general medical technology or societal development in two years (between 2015 and 2017) may have had an impact. However, the plan started at the end of 2012 is still in the same way as of 2021, and the regulations and policies established at the beginning are being maintained. Therefore, it was difficult to find changes that may affect the study findings other than the implementation of the regional trauma system. There will be limitations in complete control of such changes in a retrospective observational study. Please understand the limitation of this study design.

3. Related to the response for comment no 1, authors should explain the time lag between master plan development (2012) and actual implementation of national trauma system in detail and explain the reasons for using only two time points in the manuscript.

**Response**:

Thanks for your comments. A master plan based on the establishment of 17 regional trauma centers nationwide in South Korea was made in 2012, and 2 to 4 trauma centers have been opened every year since 2013. In 2017, 16 centers were designated, and only 9 were officially opened and operated. We believe it will take a 2-year interval to allow the emergency medical system and the emergency medical institutions to adjust to its new policies and implement new systems. Therefore, we planned a national follow-up survey every 2 years since the implementation of the national trauma system to confirm its effectiveness. Consequently, we performed a national survey on preventable trauma death rate every 2 years beginning from 2015. This manuscript was made based on the results of the first and second surveys, namely, after conducting the 2015 and 2017 surveys. The data for trauma patients, including trauma deaths, are available after at least one year has passed. Furthermore, it takes nearly a year from sampling to an analysis of panel review results. For your information, the third investigation for the 2019 trauma deaths is currently underway in 2021. Although we could extract the data of trauma patients from 2013 to 2019 when conducting this research, we just worked on 2015 and 2017 data because we just focused on the changes in trauma performance and outcomes according to the change of preventable trauma death rates in this study. These are summarized and reflected in the limitation paragraph of the Discussion section.

Minor comments

1. The resolution of all figures should be improved.

**Response**:

We improved the resolution of all figures. We apologize for this.

2. Page 7 line 125 Hope authors change the ‘Database used for the study’ to ‘Data used for the study’ and move the contents related to panel review into this section.

**Response**:

We have changed the ‘Database used for the study’ to ‘Data used for the study’ and moved the contents related to the data for the panel review into this section accordingly.

3. Page 7 line 139, More explanation is needed for the WHO “Guidelines for trauma quality improvement programmes” and its relation to panels’ decision of preventability.

**Response**:

We have added more explanation for the WHO “Guidelines for trauma quality improvement programmes (TQIP)” and its relation to panels’ decision of preventability in the Method section as follows.

The “Guidelines for TQIP” by the WHO provides how-to-do guidance on a range of different trauma quality improvement methods. These are broadly applicable to all health care institutions that care for the injured in countries at all economic levels. One or more of the methods described in this document will be directly applicable to any given institution and will enable that institution to upgrade the level of function of its existing trauma quality improvement activities. This would help strengthen the quality of trauma care and save the lives of many injured persons.

The criteria for preventability of trauma deaths were based on World Health Organization guidelines for trauma quality improvement programmes. The main factors underlying the decision regarding the preventability of trauma deaths comprised severity of injuries and appropriateness of trauma care. Definitions were as follows.

1) “Preventable trauma deaths” were deaths that could have been prevented if appropriate steps had been taken, with accompanying injuries and sequelae considered survivable. These cases had frank deviations from the standard of care that, directly or indirectly, caused the patient's death.
2) “Potentially preventable trauma deaths” were deaths that potentially could have been prevented if appropriate steps had been taken, with accompanying injuries and sequelae considered severe but survivable. These cases had some deviations from the standard of care that, directly or indirectly, caused the patient's death.
3) “Non-preventable trauma deaths” were unavoidable deaths, as accompanying injuries and sequelae were considered non-survivable even with optimal management. Evaluation and management were appropriate according to accepted standards. If the patient had co-morbid factors that were major contributors to death, such cases were considered non-preventable trauma deaths.

4. Page 8 line 162-163 More explanations are needed for ICISS and TRISS score model. (Definition, components, how they are calculated and their differences, meaning and etc). In addition, proper references should be placed.

**Response**:

We have added the explanations of ICISS and TRISS with proper references in the Method section as follows.

The ICISS took an empirical estimation approach to injury severity scoring with the formulation of the International Classification of Disease-9 survival probability (Ps). Ps is an ICD code-specific estimate of the survival probability associated with that particular injury. The traditional ICISS is calculated as the product of Ps for as many as 10 injuries and ranges from 0 (unsurvivable) to 1 (null probability of death). Other versions of the ICISS include Ps of the worst injury and independent Ps calculated on patients with isolated injuries.

The TRISS methodology, which was introduced in the 1980s, is the most widely used formula for calculating the Ps of an individual trauma patient. It calculates the probability of survival of a trauma patient by using a formula that includes the mechanism of injury (blunt or penetrating), age (older or younger than 55 years), Injury Severity Score (ISS), and Revised Trauma Score (RTS). Once the probability of survival of the individual patient is calculated, the expected survival and number of deaths for a group of patients can be determined.

5. Preventable trauma death rate from survey data for year 2015 and 2017 can be described in monthly plot. Parallel trends are expected and authors may check whether there are specific patterns among two years of monthly plot.

**Response**:

According to your suggestion, we presented the preventable trauma death rate for 2015 and 2017 in a monthly plot as the following graph. However, there was difficulty finding out parallel trends or specific patterns among two years of monthly plot.

6. Page 11 line 233-235 This sentence should move to the discussion section.

**Response**:

Thanks for the comment. We moved the sentence “Region III employed the greatest number of beds according to the population (8.5 beds/106) and the number of trauma ICU beds per the number of patients with severe trauma (7.6 beds/100) in 2017.” to the Discussion section.

7. Overall summary should be placed in the start of the discussion section.

**Response**:

We have added a paragraph for the overall summary at the start of the Discussion section according to your comment as follows.

This study aimed to evaluate the effects of the attempts to implement and establish a national trauma system in South Korea, which shows a high preventable trauma mortality rate (>30%), comparable to that of LMICs. We planned to serially conduct national surveys every two years for the purpose of reducing the preventable trauma death rate, which was the original goal when the national trauma system project was first developed at the end of 2012. In addition, the transfer status and outcome changes of trauma patients were investigated using the NEDIS, which is registered by over 400 emergency medical institutions nationwide. In this study, severe trauma patients were more concentrated in the regional trauma centers, and the preventable trauma death rate decreased by more than 10% in only 2 years after the government-led trauma system establishment. Furthermore, the overall performance and outcomes of trauma care improved in all the emergency medical institutions nationwide.

**Reviewer #2:**

The authors of the manuscript provide results and a thorough analysis of a country-wide evaluation of emergency medical services focused on trauma care in South Korea. The government is currently implementing a new, nationally regulated emergency trauma care system, which is a subject of periodic formal evaluation. The authors collect the results of the first two evaluations, both nationwide, and rigorously analyze the effectiveness of the trauma care system under development. Their analysis indicates that the new emergency care significantly improved the treatment of trauma patients, with a substantial decrease in the preventable death rate. The contributions of this manuscript are both in the methodology, data, and the evaluation of the improvement strategy South Korea is currently establishing. As such the manuscript can be of broad use to many professionals from organizing similar panels to choosing the right evaluation methodology. Finally, the improvement model can serve as a successful example to other countries, especially ones with an underdeveloped trauma care system. Therefore, I highly recommend this manuscript for publication.

Suggested minor revisions are:

1) On page 4, line 57 the authors state that 10% of people die from injury. I suggest rephrasing this sentence to stress out that it is 10% of all deaths are due to injury.

**Response**:

We have rephrased the sentence according to your suggestion.

2) On page 4, lines 69-70 the authors should consider elaborating the statement on low-quality evidence with some more details, perhaps stressing out how the evidence in their study is more reliable.

**Response**:

Thanks for this comment. Through the literature review, we noticed that many studies reported improvement in trauma performance and outcome due to establishing a trauma system. However, a recent systematic meta-analysis showed that most of the studies were cohort-based, not population-based, retrospective reviews with low-quality evidence for the effectiveness of trauma system establishment. To evaluate the outcome of trauma system establishment, many previous studies (from the 1990s to the 2000s) used the concept of preventable trauma deaths (by panel review); however, most recent studies, after the 2000s, were based on a statistical analysis of data collected from trauma centers under the established trauma system. Although most of these recent studies covered an inclusive design of trauma systems in the United States, they mainly targeted outcomes for specific cohort (pediatric or geriatric) populations or organ injury on a statewide, not a nationwide, level. Recently, few examples of the establishment of national trauma systems that started in low- and middle-income countries (LMICs), such as African or Asian countries, have been reported; however, these trauma systems are still in their infancy, not systematic, and do not provide high-quality evidence. Consequently, many studies have evaluated the effects of trauma systems, although it is challenging that they have provided low-quality evidence. Furthermore, few studies have serially and prospectively followed up the effect of a national trauma system based on a nation-led master plan for all emergency medical institutions (EMIs) across the country.

We have elaborated the statement on low-quality evidence with more details in the section by summarizing the above sentences and citing related references.

3) Authors may consider mentioning some other, similar studies, to put their work in more context. However, I think the importance of this publication is clear enough even without a more extensive review.

**Response**:

We have added some other similar studies in the context and references section.

4) The authors should improve the resolution of their figures. Figures 1 and 5 are too blurry to be read, and the remaining figures would benefit from a more sharp display (though they are readable).

**Response**:

We improved the resolution of all figures. We apologize for this.
